# Evaluation of spinal deformity and its progression in pyogenic spondylodiscitis: A retrospective MRI study of 59 cases

**DOI:** 10.1016/j.bas.2025.104204

**Published:** 2025-02-04

**Authors:** Andreas Kramer, Jonathan Neuhoff, Santhosh G. Thavarajasingam, Rebecca Sutherland, Hugh McCaughan, Benjamin Davies, Ehab Shiban, Florian Ringel, Andreas K. Demetriades

**Affiliations:** a Department of Neurosurgery, University Medical Center Mainz, Mainz, Germany; b Department of Neurosurgery, LMU University Hospital, LMU Munich, Germany; c Center for Spinal Surgery and Neurotraumatology, Berufsgenossenschaftliche Unfallklinik Frankfurt am Main, Germany; d Imperial Brain & Spine Initiative, Imperial College London, London, United Kingdom; e Department of Infectious Diseases, Western General Hospital, Edinburgh, United Kingdom; f Department of Academic Neurosurgery, Addenbroke's Hospital, Cambridge University Hospital NHS Healthcare Trust, Cambridge, United Kingdom; g Department of Neurosurgery, University Hospital of Lausitz, Cottbus, Germany; h Edinburgh Spinal Surgery Outcome Studies Group, Department of Neurosurgery, Division of Clinical Neurosciences, NHS Lothian, Edinburgh University Hospitals, Edinburgh, United Kingdom

**Keywords:** Pyogenic spondylodiscitis, Conservative treatment, Spinal deformity progression, Non-fusion

## Abstract

**Introduction:**

Pyogenic spondylodiscitis management often remains conservative without surgical intervention, yet the risk of spinal deformity under such therapy is unclear.

**Research question:**

This study explores spinal deformity progression in conservatively treated patients and identifies predictive factors for deformity advancement.

**Material and methods:**

Retrospective cohort design with radiological data analysis from 59 patients with conservatively treated pyogenic spondylodiscitis. Deformities were categorized into four progression types reflecting severity: Type 1 (progressive vertebral body edema/endplate erosion), Type 2 (Type 1 plus disc space collapse), Type 3 (vertebral body destruction/mild translation), and Type 4 (significant segmental kyphosis >20°/severe translation).

**Results:**

Among 59 patients, 66% exhibited progressive deformity over a mean follow-up of 10.75 months. The distribution of deformity progression was: Type 1 in two cases (3%), Type 2 in seven cases (12%), Type 3 in 13 cases (22%), and Type 4 in 17 cases (29%). Progression of deformity included a 92% increase in cases with segmental kyphosis >20°; and a 167% increase in cases with segmental translation. Risk factors for significant kyphosis included >50% vertebral body erosive destruction (p < 0.01) and the presence of an epidural abscess (p < 0.05). Lumbar region involvement significantly reduced the likelihood of spinal fusion at follow-up (p < 0.05). A paravertebral abscess was significantly associated with the presence of a fractured vertebrae at follow-up (p < 0.05).

**Discussion and conclusion:**

This study underscores the importance of closely monitoring patients with conservatively managed pyogenic spondylodiscitis for progressive spinal deformity, and suggests considering early surgical intervention in cases with a high risk of progression.

## Highlights

• 66% of conservatively treated pyogenic spondylodiscitis cases exhibited MRI-detected spinal deformity progression.
• Vertebral body destruction >50% significantly predicts severe spinal deformity in pyogenic spondylodiscitis.
• Lumbar spine involvement lowers the likelihood of achieving spinal fusion during follow-up.

## 1 Introduction

Pyogenic spondylodiscitis, which has a rising incidence in Western societies, presents as a severe bacterial infection affecting the intervertebral disc space and contiguous vertebral bodies (Conan et al., 2021; Thavarajasingam et al., 2023a; Kramer et al., 2023). The clinical spectrum of this condition is broad, ranging from severe back pain to debilitating neurological deficit and systemic septic states, potentially culminating in life-threatening complications (Kehrer et al., 2015).

Many healthcare professionals recommend a conservative treatment regimen for the majority of cases, typically involving intravenous and oral antibiotics, with or without immobilization, as the initial approach. In contrast, surgical intervention is often reserved for complex cases that present with neurological compromise or significant spinal instability (Bettini et al., 2009; Frederickson et al., 1978).

Due to the diverse clinical presentations, there is currently no consensus on the optimal timing for prioritizing surgical over conservative treatments for pyogenic spondylodiscitis, reflecting the variability in treatment strategies adopted internationally (Berbari et al., 2015; Kramer et al., 2024). Recent literature suggests a shift towards early surgical management, leading to potential benefits such as mortality reduction, efficient eradication of the infectious focus and correction of spinal malalignment (Thavarajasingam et al., 2023b; Lener et al., 2020; Alas et al., 2020; Neuhoff et al., 2023a, 2023b; Lin et al., 2012).

Progressive deformity is frequently cited as a justification for surgery, yet the development of spinal deformity within the context of conservative management of pyogenic spondylodiscitis remains poorly understood. There is a notable lack of evidence concerning the frequency and definition of deformity in these patients. Comprehending the implications of post-infectious spinal deformity is crucial, as it profoundly affects a patient's functional capacity and quality of life (Pellise et al., 2015; Stoop et al., 2021).

This study aims to delineate the progression of spinal deformity in conservatively managed pyogenic spondylodiscitis, with a focus on its prognostic implications, thereby contributing to the discourse on optimal treatment methodologies.

## 2 Methods

### 2.1 Study design and patient selection

This retrospective cohort study aimed to evaluate the progression of spinal deformities in patients with pyogenic spondylodiscitis treated conservatively, hypothesizing that specific imaging characteristics are predictive of deformity progression. Patients diagnosed between January 2017 and December 2022 at a collaborative database of a tertiary spinal service and a tertiary infectious diseases service, with follow-up imaging data available, were included. Baseline imaging findings and progressive spondylodiscitis were monitored through MRI scans, with an average follow-up period of 10.75 months from the initial diagnosis.

### 2.2 Data collection and accuracy

Two independent investigators, both attending neurosurgeons specialized in spine surgery, extracted data separately and compared their ratings. Discrepancies were resolved through consensus. This method ensured high data accuracy and reliability.

### 2.3 Imaging assessment

The evaluation of imaging data emphasized general baseline findings and established MRI criteria for spondylodiscitis diagnosis, focusing particularly on parameters indicative of disease progression and potential spinal instability:

• Spinal region: Categorized as cervical (C1 – C6), cervicothoracic (C7 – T2), thoracic (T3 – T10), thoracolumbar (T11 – L1), lumbar (L2 – L4), or lumbosacral (L5 – S1).
• Discitis: Inflammation or infection of the intervertebral disc, evident on MRI as a loss of disc height and structural integrity. Characterized by T2 hyperintensity and T1 hypointensity, with marked enhancement on contrast-enhanced scans, indicating increased water content and inflammation.
• Bony erosion: Presence of endplate erosion in vertebral bodies involved in spondylodiscitis, identifiable on MRI as irregularities or discontinuities of the endplate margins.
• Vertebral body destruction: Categorized by the extent of destruction as less than 50% or greater than 50% of involved vertebral bodies. On MRI, this includes loss of vertebral height and altered signal intensities on T1 (low signal) and T2 (high signal) weighted images.
• Edema: Presence of edema involving more than 50% of an affected vertebral body, indicating significant inflammatory activity within the bone and characterized on MRI by increased signal intensity on T2-weighted images and decreased signal on T1-weighted images.
• Epidural abscess: An accumulation of pus in the epidural space adjacent to the infected vertebral bodies, identifiable on MRI by its high T2 signal intensity, low T1 signal intensity before contrast, and enhancement following contrast administration.
• Paravertebral soft tissue reaction: Inflammatory changes in the tissues surrounding the vertebrae, characterized on MRI by contrast enhancement of the soft tissues adjacent to the infected vertebral bodies, without a defined collection indicative of an abscess.
• Paravertebral abscess: Localized accumulation of infected material adjacent to the vertebral bodies, identifiable on MRI scans by its high T2 signal intensity, low T1 signal intensity, and characteristic rim enhancement post-contrast administration.
• Kyphosis: Defined as a curvature of the spine with a segmental angle greater than 20°, measured using the Cobb method on sagittal MRI images.
• Scoliosis: Identified as a lateral curvature of the spine exceeding 10° on segmental level, also measured using the Cobb method, but on coronal MRI images.
• Translation: Assessed by the Meyerding grading system, which quantifies horizontal misalignment of vertebral bodies on MRI, indicating spinal instability or degeneration.
• Subluxation: Partial dislocation of a vertebra related to spondylodiscitis, visible on MRI as misalignment on sagittal or axial views, indicative of ligamentous compromise or infection-induced degenerative changes.
• Interbody Fusion: Fusion in MRI imaging was defined as the absence of a fluid signal within the intervertebral disc space on T2-weighted images and the simultaneous presence of continuous bridging bone marrow signal across the adjacent vertebral bodies on T1-weighted images. Spinal ankylosis, characterized by ossification of the posterior elements without evidence of interbody fusion, was not rated as fusion.
• Fractures: Vertebral fractures were assessed on MRI, defined by a vertebral height loss exceeding 20% compared to the initial imaging of the same vertebra.

### 2.4 Classification of deformity progression

**Fig. 1:**
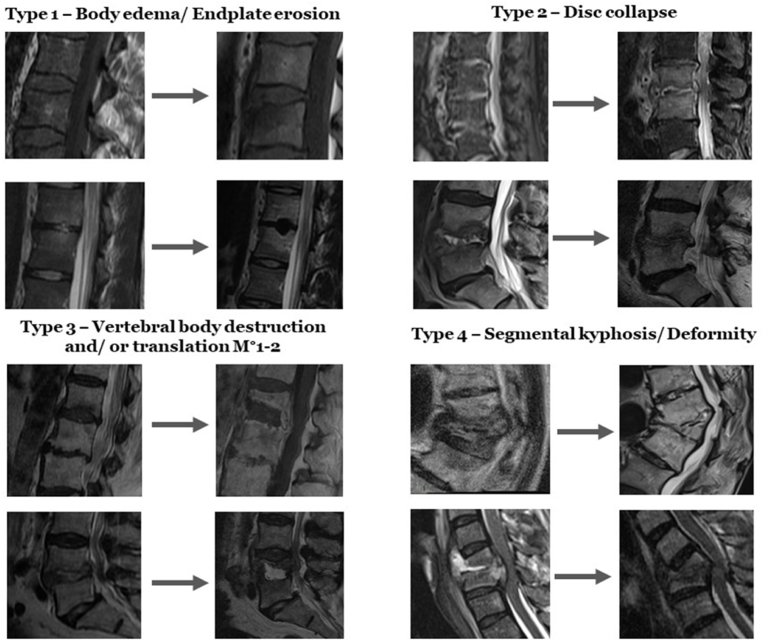
Progression Types in Conservatively Treated Pyogenic Spondylodiscitis. The figure showcases the classification of imaging progression in conservatively treated pyogenic spondylodiscitis, captured through MRI sequences over time. Type 1 demonstrates the initial stages of vertebral body edema and endplate erosion, followed by Type 2 which shows additional disc space collapse. Type 3 illustrates further progression with evident vertebral body destruction and/or mild anterior or posterior translation, classified as Meyerding Grade 1–2. The most advanced stage, Type 4, is characterized by a significant kyphotic deformity of the spinal segment. The chronological sequence is indicated by arrows pointing from the initial to the follow-up MRI images, highlighting the natural evolution of the condition without surgical intervention.

Based on the assessment of the parameters mentioned above, we developed a classification system to categorize progressive spinal deformity into four types, each reflecting the severity of the disease and the potential for segmental instability. This stratified approach was specifically designed for this study to evaluate the cumulative nature of structural changes as the disease progresses, with each type representing a stage in the course of pyogenic spondylodiscitis. Fig. 1 illustrates these progression types, demonstrating the evolution from initial to follow-up MRI sequences.

#### 2.4.1 Type 1: Progressive vertebral body edema and/or endplate erosion

This type represents the progression of early inflammatory response, characterized by increasing edema within the vertebral body and erosion of the affected endplates. These changes indicate the beginning of structural compromise but may not yet significantly impact spinal stability.

#### 2.4.2 Type 2: Type 1 with addition of disc collapse

Progressing from Type 1, this stage includes the collapse of the disc space, which is a direct consequence of the infection weakening the intervertebral disc. This collapse results in a reduction of disc height and may begin to affect spinal alignment and stability.

#### 2.4.3 Type 3: Vertebral body destruction and/or mild translation

In this more advanced stage, there is significant structural progression of the disease, resulting in the destruction of the vertebral body, exceeding 50% loss of vertebral height. In addition, mild translation of the vertebral bodies (Meyerding° 1) may be present, indicating increased instability and potential for significant deformity.

#### 2.4.4 Type 4: Severe segmental kyphosis or translation

The most severe form of progressive MRI findings encompasses substantial deformities with either progressive (additional 5° of segmental kyphosis at follow-up) or newly diagnosed segmental kyphosis exceeding 20° compared to the baseline status. Patients with newly diagnosed severe translation of vertebral bodies (≥Meyerding° 2) at follow-up were rated in the same category. These changes represent a critical level of spinal instability and deformity.

### 2.5 Statistical analyses

Logistic regression models were applied to identify predictive factors for outcomes such as progressive deformity, translation, and fracture risk, considering relevant imaging variables upon admission. Our analyses focused on identifying risk factors for the development of progression as defined by the progression types and on finding associations between single imaging outcomes and baseline characteristics. To enhance the robustness of our findings, we employed covariate boosting techniques and checked covariates for multicollinearity. In this context, variable importance analysis using Elastic Net, LASSO, and Ridge regression models was performed to determine the significance of predictors. Model diagnostics and validation involved assessing the fit and predictive power through residual deviance, standard errors, and p-values. This comprehensive approach combined detailed imaging evaluation with advanced statistical analyses to identify significant predictive factors for the development of spinal deformity in conservatively treated pyogenic spondylodiscitis patients.

## 3 Results

### 3.1 Descriptive analyses of baseline imaging characteristics

A detailed summary of the imaging findings at baseline is presented in Table 1. This table outlines the prevalence of various imaging characteristics observed in the patient cohort at initial presentation.

**Table 1:** Distribution of baseline parameters measured using the first MRI study at the initial diagnosis of de novo spinal infection.

Imaging Characteristic	Baseline (n, % of Patients)
Spinal Region	
- Cervical	5 (8.47%)
- Cervicothoracic	1 (1.69%)
- Thoracic	12 (20.34%)
- Thoracolumbar	6 (10.17%)
- Lumbar	27 (45.76%)
- Lumbosacral	8 (13.56%)
Bony Erosion	48 (81.36%)
Vertebral Body Destruction <50%	12 (20.34%)
Vertebral Body Destruction >50%	18 (30.51%)
Edema Vertebral Body >50%	52 (88.14%)
Discitis	58 (98.31%)
Epidural abscess	32 (54.28%)
Paravertebral soft tissue reaction	42 (71.19%)
Paravertebral abscess	18 (30.51%)
Segmental kyphosis >20°	12 (20.34%)
Translation	3 (5.08%)

Bony erosion was observed in a majority of 48 (81.36%) cases, indicating its prevalent nature in pyogenic spondylodiscitis. As a matter of course, the intervertebral disc was affected in almost all cases, 58 (98.31%). Bony destruction of the vertebral body varied, with less than 50% involvement in 12 (20.34%) patients and destruction of more than 50% in 18 (30.51%). A significant majority of 52 (88.14%) cases, exhibited edema of more than 50% of the affected vertebral bodies. An epidural abscess was identified in over half of the cases, 32 (54.24%), resulting in spinal canal stenosis in 22 (37.29%) of patients. Paravertebral soft tissue reaction and abscess were present in 42 (71.19%) and 18 (30.51%) of cases, respectively. Despite these widespread pathological changes, normal spinal alignment upon admission was maintained in 40 (67.80%) of the patients.

### 3.2 Follow-up imaging characteristics

Imaging follow-up was conducted using MRI, with an average follow-up of 10.75 months from the initial diagnosis. Table 2 provides follow-up data, showing the changes observed in these imaging features over time, providing a comparative perspective on the progression of deformity.

**Table 2:** Imaging follow-up parameters extracted from the most recent available MRI of each patient.

Imaging Characteristic	Follow-up n (% of Patients)	Percentage Change (%)
Segmental kyphosis >20°	23 (38.98%)	92%
Translation	8 (13.56%)	167%
Subluxation	0 (0%)	0%
Fusion	22 (37.29%)	–
Progressive Deformity	39 (66.1%)	–
Fracture	9 (15.25%)	–

At this follow-up, a significant number of patients, 39 (66.10%), exhibited progressive deformity, highlighting the high rate of overall progression in this condition. The incidence of segmental kyphosis greater than 20° increased from an initial 12 cases (20.34%) to 23 cases (38.98%), representing a 91.67% increase. Similarly, translation, initially present in three (5.08%) cases, rose to eight cases (13.56%) at follow-up, marking a substantial increase of 166.67%. Notably, spinal fusion, as defined above, was achieved in only 22 (37.29%) of the cases, revealing a relatively low rate of this crucial healing process.

### 3.3 Type of progression at follow-up

**Fig. 2:**
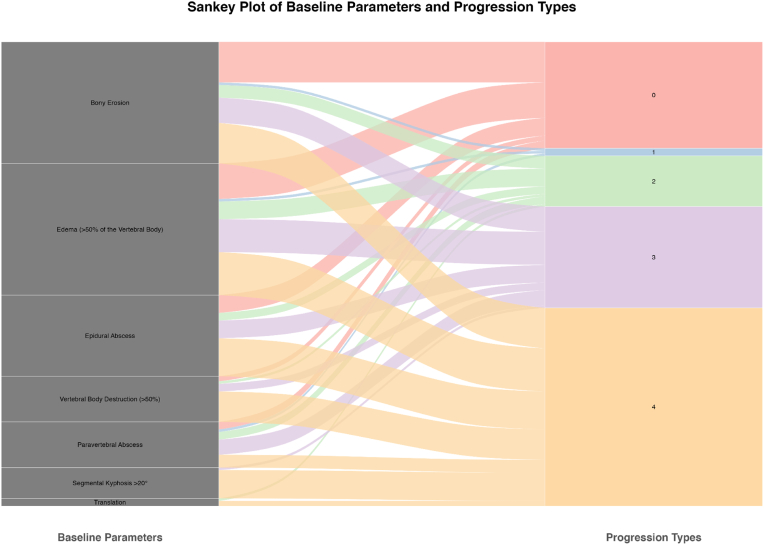
Sankey Plot of Baseline Parameters and Progression Types in Pyogenic Spondylodiscitis This figure illustrates the flow and relationships between initial imaging characteristics and subsequent deformity progression types in conservatively treated pyogenic spondylodiscitis. The left side of the plot displays baseline parameters, while the right side categorizes patients into progression types. '0′ indicates no progression of deformity compared to baseline, and '1′ through '4′ represent the progression types as defined in the manuscript. Lines connecting the parameters to the progression types show transition probabilities, with the thickness of the lines indicating the relative number of cases transitioning from each parameter to each progression type.

Observed progression types at follow-up demonstrated a range from no change to severe deformity. These findings are visualized in a Sankey plot in Fig. 2. No progression was identified in 20 cases (33.90%). Type 1 progression, featuring progressive vertebral body edema and/or endplate erosion, was noted in two cases (3.39%). Progression to Type 2, which involves the addition of disc collapse, was observed in seven cases (11.86%). Type 3 progression, signifying vertebral body destruction and/or mild translation, appeared in 13 cases (22.03%). The most advanced, Type 4 progression, indicating significant segmental kyphosis or severe translation, was found in 17 cases (28.81%).

### 3.4 Statistical associations and predictors of deformity progression

Our multivariate regression analyses identified a significantly increased risk for the development of a severe spinal deformity, classified as Progression Type 4, with vertebral body destruction greater than 50% (OR = 13.70, p < 0.01) and the presence of an epidural abscess at baseline (OR = 13.50, p < 0.05, 95%). These findings highlight the substantial impact of these factors on the risk of severe spinal deformity progression. Bony erosion of the endplates alone was not a significant predictor for severe spinal deformity at follow-up, and no baseline imaging variable was found to significantly predict progression to Types 1–3 in our cohort (Fig. 2).

Involvement of the lumbar spine was significantly associated with a reduced likelihood of achieving spinal fusion at follow-up (OR = 0.18, p < 0.05), indicating potential regional variances in the progression and healing course of the disease, particularly disadvantaging this mobile region of the spine.

Regarding the occurrence of translation at follow-up, our analyses indicated a notable negative association with the presence of paravertebral soft tissue reaction (OR = 0.0625, p < 0.05). This suggests that patients who initially exhibited paravertebral soft tissue reactions without evident formation of an abscess were less likely to develop spinal translation during the follow-up period. Fractures at follow-up were significantly associated with the presence of a paravertebral abscess (OR = 6.85, p < 0.05).

These observations emphasize the importance of closely monitoring patients with extensive vertebral body involvement, as it significantly correlates with the progression of deformity, underscoring the need for potentially adjusting treatment plans based on these initial MRI assessments. The detailed statistical associations are summarized in Table 3.

**Table 3:** Results of the multivariate regression model analyzing predictors for developing severe deformity, as outlined by a type 4 progression. This table also includes other significant associations between baseline parameters and follow-up outcomes.

Predictor	OR	p-value	Association with:
Vertebral Body Destruction >50%	13.70	<0.01	Severe deformity (Type 4)
Epidural Abscess at Baseline	13.50	<0.05	Severe deformity (Type 4)
Involvement of Lumbar Spine	0.18	<0.05	Absence of fusion at FU
Paravertebral Soft Tissue Reaction	0.0625	<0.05	Negative association with translation
Paravertebral Abscess	6.85	<0.05	Fracture at follow-up FU

## 4 Discussion

This study offers a detailed examination of progressive spinal deformity in patients with pyogenic spondylodiscitis managed conservatively. Our retrospective analysis demonstrates a pronounced progression in segmental kyphosis and translational deformity, evidenced by a 92% increase in kyphotic deformation and a 167% increase in translation, upon follow-up. These findings indicate a significant trend towards segmental deterioration in pyogenic spondylodiscitis, signaling an inherent risk of structural compromise that could detrimentally impact long-term patient functionality and quality of life.

Critically, vertebral body involvement or destruction exceeding 50% at initial presentation emerges as a pivotal predictor for developing spinal deformity. This emphasizes the importance of structural integrity in determining the likely success of conservative measures in preserving spinal alignment. Given the strong association between substantial vertebral body involvement and adverse imaging outcomes, there is a compelling argument for re-evaluating the efficacy of traditional conservative strategies, like bracing and bedrest, particularly in cases with significant vertebral damage.

### 4.1 Management strategies and risk of progressive deformity

Classical conservative management has suggested that immobilization, through bedrest or bracing, could mitigate the risk of spinal deformity (Frederickson et al., 1978). Despite this, there is no high-quality evidence supporting the effectiveness of such measures in preventing deformity during the conservative treatment of pyogenic spondylodiscitis. Our study sheds light on the drawbacks of these traditional strategies, highlighting the potential for adverse imaging outcomes and the significant progression of spinal deformity in conservatively managed cases. Bettini et al. reported the success of conservative treatment, including the use of spinal bracing, yet did not provide concrete evidence regarding the prevention of progressive deformity (Bettini et al., 2009). Apart from external measures like bracing, achieving rapid fusion of the affected spinal segments is a critical objective in both surgical and conservative management strategies. It is often believed that spontaneous fusion is a frequent occurrence in the natural post-inflammatory course of the disease. However, this assumption lacks robust empirical support, and the evidence for a high spontaneous fusion rate in conservatively managed cases remains to be substantiated. Contrary to these assertions and consistent with a reported fusion rate of 35% by Frederickson et al. (1978), our findings indicate a relatively high incidence of non-fusion at follow-up, suggesting this could be a pivotal factor in the development of progressive deformity and persisting symptoms.

These findings raise significant doubts about the ability of conservative measures to effectively prevent progressive spinal deformity in the treatment of pyogenic spondylodiscitis. The high rate of non-fusion and deformity progression in our cohort underscores the urgency to re-evaluate these strategies, particularly their role in controlling spinal malalignment.

In their study on the role of follow-up MRI findings in conservatively managed spondylodiscitis patients, Kowalski et al. reported that 50% of patients with equivocal or worsened imaging findings exhibited no clinical improvement or even experienced a worsening of clinical symptoms at follow-up (Kowalski et al., 2006). This underscores the clinical significance of deformity progression, pointing to the necessity for further investigation into the correlation between imaging and clinical outcomes.

As minimal invasive surgical stabilization techniques and devices constantly evolve, they might offer a more definitive treatment of an existing or potential deformity and therefore enable a more satisfactory long-term outcome for this disease, the incidence of which is constantly increasing (Schwendner et al., 2023; Viezens et al., 2017). In addressing pyogenic spondylodiscitis, the decision-making process in clinical practice should be guided by careful consideration of the potential risks associated with both conservative and surgical treatment approaches. Our findings emphasize the significant risks of progressive deformity and non-fusion inherent in conservative management. Concurrently, surgical interventions, while potentially mitigating these risks, are not without complications, such as implant failure and adjacent level disease (Abboud et al., 2023). This necessitates a nuanced approach, where treatment decisions are tailored based on disease severity and a patient's overall health status. The focus should be on balancing the long-term benefits and risks inherent to each treatment modality. Further research is essential to deepen our understanding of the effectiveness of these approaches, particularly in their capacity to enhance overall patient outcomes and quality of life.

### 4.2 Limitations and future directions

The insights from this study on the progression of spinal deformity in conservatively treated pyogenic spondylodiscitis, while significant, are tempered by several limitations. Primarily, the retrospective nature of this analysis introduces the potential for selection and information biases, underscoring the necessity for prospective studies for confirmation and expansion of our findings. The study's execution within two tertiary hospitals may limit the generalizability of results to different geographic or healthcare settings, suggesting the need for multicenter research. A notable limitation is the absence of a comparative group undergoing surgical treatment, restricting our capacity to ascertain the relative effectiveness of conservative versus surgical modalities.

Significantly, this investigation's focus was on deformity progression rather than direct clinical outcomes, which are pivotal for comprehensively assessing patient well-being and the effectiveness of treatment strategies. Deformity assessment in this study was based on MRI as imaging modality. It is acknowledged that a comprehensive assessment of spinal deformity requires standing and functional X-Ray imaging, which was not routinely in the study center. As such, this data is lacking for the investigated cohort. However, it must be assumed, that the true rate and extent of deformity may be even higher when additional upright imaging modalities are incorporated, which will be a subject to future research.

Furthermore, the emphasis on imaging data without concurrently evaluating nor correlating to patient-reported outcomes might not fully capture the impact of spinal deformities on a patient's quality of life. Additionally, the study's relatively brief follow-up period might not fully reflect the disease's long-term trajectory, pointing to the need for extended observation in future studies.

## 5 Conclusion

This study illuminates the considerable risk of progressive spinal deformity in conservatively managed patients with pyogenic spondylodiscitis. It underscores the importance of adopting a dynamic, multidisciplinary approach to patient management; and of potentially identifying patients at risk of developing a relevant spinal deformity, who might therefore benefit from a more proactive and early surgical management. Our data suggest that regular follow-up should be integral to the treatment process, allowing for timely adjustments in the therapeutic strategy as the patient's condition evolves.

## Informed consent and patient details

This study includes anonymized MRI data, which was used in accordance with the informed consent obtained as part of the treatment contract with the treating clinic.

## Contributors

Andreas Kramer: Contributed to the conception and design of the study, acquisition of data, and analysis and interpretation of data. Drafted significant portions of the manuscript and critically revised it for important intellectual content. Provided final approval of the version to be submitted.

Jonathan Neuhoff: Contributed to the conception and design of the study, acquisition of data, and analysis and interpretation of data. Played a major role in drafting the manuscript and revising it critically for important intellectual content. Provided final approval of the version to be submitted.

Santhosh G. Thavarajasingam: Contributed to the analysis and interpretation of data. Assisted in drafting sections of the manuscript and provided critical revisions. Approved the final version of the manuscript.

Benjamin Davies: Contributed to the analysis and interpretation of data, provided key intellectual input during revisions, and approved the final version of the manuscript.

Ehab Shiban: Provided critical revisions to the manuscript for important intellectual content. Contributed to data interpretation and approved the final version of the manuscript.

Florian Ringel: Contributed to the study's conception and design, and played a key role in revising the manuscript critically for important intellectual content. Provided final approval of the version to be submitted.

Andreas K. Demetriades: Involved in the conception and design of the study, and provided significant contributions to the manuscript through critical revisions and intellectual content. Approved the final version of the manuscript.

All authors have read and approved the final manuscript, agreeing to be accountable for all aspects of the work.

## Use of AI in the writing process

During the preparation of this work, the authors used ChatGPT by OpenAI for language editing to improve the readability of the manuscript. After using this tool, the authors reviewed and edited the content as needed and take full responsibility for the content of the publication.

## Funding

No funding was provided for the conduct of this research or the preparation of this article. The study design, data collection, analysis, interpretation, writing of the report, and decision to submit the article for publication were carried out independently by the authors without any financial support or involvement from external sponsors.

## Declaration of competing interests

The authors have no financial or personal relationships with other people or organizations that could inappropriately influence (bias) their work.
